# Intermittent fasting and exercise therapy abates STZ‐induced diabetotoxicity in rats through modulation of adipocytokines hormone, oxidative glucose metabolic, and glycolytic pathway

**DOI:** 10.14814/phy2.15279

**Published:** 2022-10-28

**Authors:** Ejime A. Chijiokwu, Eze K. Nwangwa, Mega O. Oyovwi, Alexander O. Naiho, Victor Emojevwe, Ejiro P. Ohwin, Prosper A. Ehiwarior, Evelyn T. Ojugbeli, Udoka S. Nwabuoku, Onome B. Oghenetega, Ofulue O. Ogheneyoma

**Affiliations:** ^1^ Department of Physiology Faculty of Basic Medical Science College of Health Sciences Delta State University Abraka Delta State Nigeria; ^2^ 524172 Department of Human Physiology Achievers University Owo Ondo State Nigeria; ^3^ Department of Physiology University of Medical Sciences Ondo Ondo State Nigeria; ^4^ Department of Medical Biochemistry Faculty of Basic Medical Science College of Health Sciences Delta State University Abraka Delta State Nigeria; ^5^ Department of Physiology Faculty of Basic Medical Science Babcock University Ilisan‐Romo Ogun State Nigeria

**Keywords:** adipocytokines, Diabetes, exercise, intermittent fasting, metabolic enzymes, glycolytic enzymes

## Abstract

Diabetes is a global, costly, and growing public health issue. Intermittent fasting (IF) and exercise therapy have been shown to improve insulin sensitivity (IS) in large studies, although the underlying processes are still unknown. The goal of this study, which included both nondiabetic and diabetic rats, was to look at the mechanisms of intermittent fasting and exercise in the management of diabetotoxicity. The effects of starvation and honey on the oral glucose tolerance test, insulin tolerance test, adipocytokines, oxidative glucose metabolic enzymes, glycolytic enzymes, food intake, and body weight in rats with streptozotocin‐induced diabetes were also investigated. In the nondiabetic phase, rats were administered an oral regimen of distilled water (0.5 ml/rat), honey (1 g/kg body weight), and interventions with IF, and starvation for 4 weeks while in the diabetic phase, after STZ or citrate buffer injections, interventions with IF, exercise, starvation, and honey treatment began for 4 weeks. At all OGTT and ITT points, there was a substantial rise in glucose in the STZ group. Adipocytokines hormone, oxidative glucose metabolic enzymes, glycolytic enzymes, and body weight were all affected by STZ when compared to starvation and honey, however, IF and exercise significantly reduced these alterations. In diabetic rats, intermittent fasting and exercise enhanced serum adipocytokines levels. These findings imply that adipokines modulate glycolytic/nonmitochondrial enzymes and glucose metabolic/mitochondrial dehydrogenase to mediate the antidiabetic effects of intermittent fasting and exercise.

## Abstract 2

Intermittent fasting and exercise therapy abates STZ‐induced diabetotoxicity in rats through modulation of adipocytokines hormone, oxidative glucose metabolic, and glycolytic pathway.

## 1 INTRODUCTION

Fasting hyperglycemia decreased insulin secretion, and insulin receptor insensitivity are all symptoms of diabetes mellitus, a metabolic condition (Hudish et al., 2019). Diabetes mellitus is reported to impact more than 100 million individuals worldwide and is one of the world's top five causes of death (Otovwe & Akpojubaro, 2020; Yang et al., 2019). Persistent hyperglycemia in diabetics has been shown to generate excessive reactive oxygen species (ROS) generation in many organs by glucose auto‐oxidation and/or protein glycation (Saddala et al., 2013). In animal models and humans with diabetes, there have also been findings of altered antioxidative enzyme activity and enhanced lipid peroxidation (Kade et al., 2008; Prabakaran & Ashokkumar, 2013; Schmatz et al., 2012). This condition is the result of issues associated with modern lifestyles, such as a high intake of processed foods, a growing geriatric population, decreased physical exercise, and obesity (Bekele et al., 2020). Type 2 diabetes mellitus (T2DM), cardiovascular diseases (CVDs), and fatty liver disease are all more common in people who have metabolic syndrome (Daryabor et al., 2019).

Food restriction (FR) is defined as a reduction in food intake while maintaining minimal nutritional levels. In humans with T2DM (Albosta & Bakke, 2021) and animal models, it has already shown improvements in pancreatic beta‐cell activity, blood glucose control, and other parameters (Alejandra et al., 2018; Elesawy et al., 2021). Some of the various approaches utilized to create FR is intermittent fasting (IF) (Kunduraci & Ozbek, 2020) and starvation. Hypoglycemia, ketoacidosis, dehydration, hypotension, and thrombosis have all been linked to diabetics who practice IF (Hu et al., 2019). Short‐term starvation causes insulin resistance in humans, according to previous research (Wang et al., 2020). More interestingly, exercise and intermittent fasting have long been recognized as important non‐pharmacological tools for the treatment of diabetes (Corley et al., 2018; Harvie & Howell, 2017; Sampath Kumar et al., 2019; Sutton et al., 2018) and accepted as adjunctive therapy in the management of type 2 diabetes mellitus owing to their ability to improve insulin sensitivity and insulin‐stimulated muscle glucose uptake, both of which improve glucose utilization (Ko et al., 2018). As a result, elucidating the mechanisms underlying this type of intermittent fasting and exercise therapy‐related improved insulin sensitivity could help researchers to better understand how insulin sensitivity develops in conditions like obesity and type 2 diabetes.

## 2 MATERIALS AND METHODS

### 2.1 Animal use and handling

All animal experiments were conducted in accordance with protocols authorized by the Research Committee on the Ethical Use of Animals (DSUA Care), Reference no REC/FBMS/DELSU/21/121. The researchers employed 80 adult Sprague Dawley rats of similar age (10–12 weeks) and weight (180–250 g) for the investigation. For 2 weeks of acclimation, the rats were kept in a regular habitat with uniform husbandry and photoperiodic conditions (12 h of light and 12 h of darkness) and an ambient room temperature of 280°C–300°C. Throughout the trial, all of the rats were kept in clean wooden cages with unlimited access to water and a standard rat chow diet. The National Research Council's ‘Guide for the Care and Use of Laboratory Animals (NRC, 2011) was utilized to ensure that the animals used in this investigation were treated as humanely as possible.

### 2.2 Chemicals

Commercially available honey (Golden Glory, Australia) was bought from a local market in Abraka, Delta State. Honey (1 g/kg/day) was diluted with distilled water before being given to the rats by gavage. Streptozotocin (STZ, 99% purity) was supplied by Sigma‐Aldrich. All other chemicals used were analytical grade and also were obtained from Sigma‐Aldrich.

### 2.3 Induction of diabetes

Diabetes was induced by giving a single intraperitoneal injection of low dose streptozotocin (STZ, 50 mg/kg b.w.) in freshly made 0.1 M citrate buffer (pH 4.5). To avoid hypoglycemia, these rats were given unrestricted access to standard rats’ chow during the night after being injected with streptozotocin in a solution of saccharose (10 g/100 ml). Diabetes was diagnosed 72 h after STZ injection in rats with a fasting blood glucose level of more than 200 mg/dl. This was done with the One Touch UltraEasy Blood Glucose Monitoring System and a glucometer after blood was expressed from the tail vein. The nondiabetic groups were administered intraperitoneal injection of freshly made 0.1 M citrate buffer (pH 4.5) without STZ. Four weeks after STZ or citrate buffer injections, the treatments began.

### 2.4 Drugs and their preparations

Honey (Aamri & Ali, 2017; Erejuwa et al., 2011) and STZ (Aamri & Ali, 2017) dosages were determined based on past dose–response effects and early investigations, and distilled water dose was chosen based on a previous study (Aamri & Ali, 2017).

### 2.5 Experimental design

A total of 66 rats were divided into two phases: non‐diabetic and diabetic. The nondiabetic phase is divided into five groups whereas the diabetic phase is divided into six groups consisting of six rats (*n* = 6) per group. The groups of the nondiabetic phase include Nondiabetic control, Intermittent fasting, Starvation, and honey (1 g/kg body weight) groups. The groups of the diabetic phase include Control, Diabetic control, Diabetic and intermittent fasting, Diabetic and starvation, Diabetic and Exercise, and Diabetic and honey (1 g/kg body weight) groups.

### 2.6 Physiological intervention approach

#### 2.6.1 Intermittent fasting intervention

The intermittent fasting (IF) group was given absolute food deprivation for 24 h, followed by ad libitum access to rat chow for another 24 h. At noon, the IF group's food was withdrawn or made available. For the duration of the experiment, the IF group had unlimited access to water. Bodyweight change and food intake were tracked throughout the study.

#### 2.6.2 Starvation intervention

For 2 weeks, rat chow was withheld from a group of rats to test the effect of starving (Namazi et al., 2016). During the protracted hunger, none of the rats died.

#### 2.6.3 Exercise intervention

Individual cages with a running wheel (Accelerator Ltd.) were used to house the exercising animals, who had free access to the wheel for 24 h a day (method of Szalai et al., 2014). The exercising protocol was chosen to isolate the effects of exercising from the additional stress associated with forced exercise regimens. It is defined as a voluntary wheel‐running paradigm. The average running distance permitted throughout the exercising time was 4 km/day/animal for uniformity.

### 2.7 Measurement of body weight and food intake

The rats’ body weight was measured and documented at the start of the trial, and they were then weighed weekly with a digital weighing scale to see how much they had changed. The pancreas, liver, and heart's relative organ weights were also measured and recorded. Furthermore, daily feed intake was assessed and recorded in percentage. First, the total weight of feed provided per group was subtracted from the weight of daily feed remnants.

### 2.8 Sample collection and preparation

Rats were euthanized following an overnight fast under diethyl ether anesthesia at the end of the fourth week (28 days) of treatment. Fasting blood glucose, insulin concentration, glucose tolerance and insulin tolerance tests, glucose intolerance and insulin sensitivity, adipocytokines hormones (adiponectin, ghrelin, resistin, and irisin), oxidative glucose metabolic enzymes, and adipocytokines hormones (adiponectin, ghrelin, resistin, and irisin) were all tested (ICDH, SDH, G6PDH, and LDH). Following that, liver tissues were dissected, cleaned of adherent tissues, washed with physiological saline containing 0.9 percent (w/v) cold normal saline, and pat dried on filter paper. The tissues were homogenized in a Teflon homogenizer (Heidolph Silent Crusher M) and then centrifuged at 10,000*g* for 15 min at 4°C. The activity of glycolytic/mitochondrial enzymes (G6Pase, F1,6BPase, HKase, and PKase) was evaluated by using a spectrophotometer to measure the absorbance of the samples (Shimadzu UV 1700).

## 3 BIOCHEMICAL ASSAY

### 3.1 Oral glucose tolerance test (oGTT) and insulin tolerance test (ITT)

The procedures from Cummings et al. (2014) were used for oGTT and ITT.

#### 3.1.1 Oral glucose tolerance test (oGTT)

The rats were subjected to a 12‐h overnight fast in the final week of treatment. Then, using a tail snip, blood was obtained for glucose measurement (time 0) on a glucometer (FreeStyle Potium Neo). The animals were then given a glucose solution of 2 g/kg per body weight via gavage, and blood glucose concentrations were recorded at 0, 30, 60, and 120 min.

#### 3.1.2 Insulin tolerance test

After the OGTT, an insulin tolerance test (ITT) was performed 48 h later. The animals were subjected to a 4‐h food restriction in this case. The blood was then drawn from the animal's caudal end and used to measure glucose (time 0) using a glucometer (FreeStyle Potium Neo). After that, the animals were given an intraperitoneal injection of ordinary human insulin (Humulin) at a dose of 0.75 U/kg per body weight, and blood glucose levels were monitored at 0, 30, 60, and 120 min.

### 3.2 Determination of adipocytokines (adiponectin, ghrelin, irisin, and resistin)

The levels of adipocytokines (Adiponectin, ghrelin, irisin, and resistin) were tested using rat adiponectin, ghrelin, irisin, and resistin ELISA kit, as described by Jiménez‐Maldonado et al (2019). Serum samples diluted to 1:500 were used in the experiment. Within the first 30 min after the stop solution was applied, absorbance was measured at 450 nm (MyBiosource, Inc.). The mean absorbance of the samples was computed after they were tested in duplicate. The adiponectin and ghrelin/irisin assays have sensitivity limits of 0.4 ng/ml and 0.4 pg/ml, respectively, and quality control was verified using the kit's standards.

### 3.3 Estimations of oxidative glucose metabolic and glycolytic enzymes in rat serum

Using the ELISA approach, the oxidative glucose metabolic status (ICDH, SDH, G6PDH, and LDH) and glycolytic enzyme activities (G6Pase, F1,6BPase, HKase, and PKase) in serum and liver were examined and quantified (R & D systems, USA and Thermo Fisher Scientific, respectively).

### 3.4 Statistical analysis

Graph pad prism 8 Biostatistics software was used to examine the data (Graph pad Software, Inc., version 8.0). All data were reported as the mean standard error of the mean (SEM). Following that, a one‐way analysis of variance (ANOVA) was used, followed by a post hoc test (Bonferroni) for multiple comparisons. The significance level for all tests was set to *p* < 0.05.

## 4 RESULTS

### 4.1 Effect of intermittent fasting, starvation, exercise and honey on oral glucose tolerance test (OGTT) in naïve, and streptozotocin‐induced diabetes in levels in male rats

**FIGURE 1:**
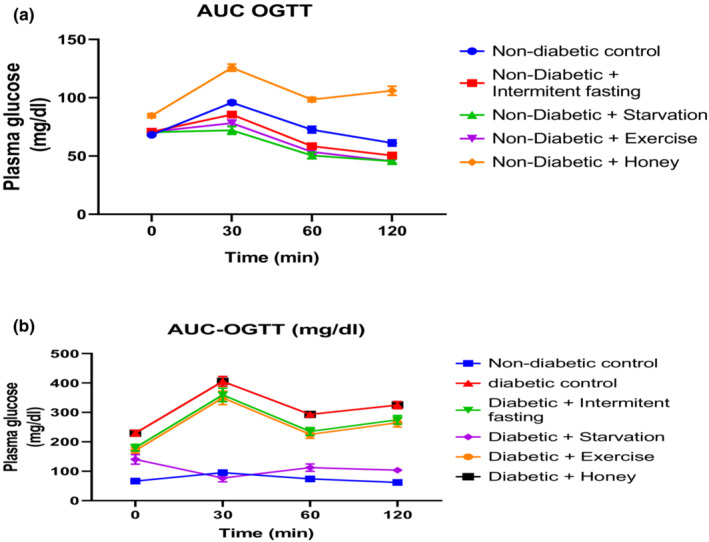
(a) Area under the curve (AUC) of glucose tolerance test on normal and different intervention protocols. (b) AUC of glucose tolerance test on normal, diabetic rats and different intervention protocols

The effect of intermittent fasting, starvation, Exercise and honey on OGTT in naïve and streptozotocin‐induced Type 2 Diabetes Mellitus (T2DM) in male rats is shown in Table 1a/Figure 1a and Table 1b/Figure 1b. This OGTT was used to evaluate glucose metabolism in nondiabetic and diabetic rats. The result of the OGTT of intervention with intermittent fasting, starvation. Exercise in rats shows a decrease in blood glucose levels at all points of the OGTT when compared with that of the nondiabetic control rats as represented in Table 1a and Figure 1a whereas rats treated with honey revealed a significant increase in blood glucose levels at all point of the OGTT relative to intermittent fasting, exercise, starvation, and nondiabetic control rats.

**TABLE 1:** (a) Glucose tolerance test on normal and different intervention protocols. (b) Glucose tolerance test on normal, diabetic rats and different intervention protocols

Groups	Plasma glucose (mg/dl)			
	0 min	30 min	60 min	120 min
**(a)**				
Normal diabetic control	68.2 ± 3.93	95.8 ± 5.63	72.6 ± 6.11	61.2 ± 7.14
ND + Intermittent fasting	70.8 ± 5.41^*d^	85.6 ± 7.34^*cd^	58.4 ± 3.97^*cd^	50.4 ± 5.42^*cd^
ND + Starvation	70.4 ± 6.31^*d^	72.0 ± 4.40^*bd^	50.4 ± 4.17*^b^	45.6 ± 2.14^*bcd^
ND + Exercise	70.8 ± 5.43^*d^	78.2 ± 6.29^*bcd^	53.6 ± 3.54*^bcd^	45.6 ± 3.12^*bd^
ND + Honey	84.6 ± 3.95^*^	125.8 ± 5.84^*bcd^	98.4 ± 7.22^*^	106 ± 12.4^*^
**(b)**				
Normal diabetic control	66.5 ± 3.46	95.3 ± 4.39	73.8 ± 2.43	61.7 ± 3.30
Diabetic control (DC)	229.3 ± 15.31^*^	405 ± 14.36^*^	293 ± 11.31^*^	324.7 ± 3.69^*^
ND + Intermittent fasting	180 ± 8.24^ad^	359.5 ± 10.01^ad^	235.2 ± 10.22^ad^	274.8 ± 4.62^ad^
ND + Starvation	140 ± 5.34^abcd^	76.7 ± 8.30^abcd^	112.2 ± 11.00^abcd^	103.5 ± 4.14^abcd^
ND + Exercise	170 ± 6.34^ab^	349.5 ± 9.47^ab^	225.2 ± 9.62^ab^	264.8 ± 7.20^ab^
ND + Honey	229.3 ± 12.56	405 ± 9.99	293 ± 9.34	324.7 ± 8.52

Note

^a^ Showing glucose tolerance test on normal and different intervention protocol result represent Mean ± S.E.M. (*n* = 6) (One‐way ANOVA followed by Benferroni post hoc test). *P < 0.05, relative to controls. ^b^ *p* < 0.05 relative to intermittent fasting group; ^c^ *p* < 0.05 relative to Exercise group. ^d^ *p* 〈 0.05 relative to Honey group.

The results of the oral glucose tolerance test (OGTT) of streptozotocin‐induced diabetic rats showed a significant increase in blood glucose levels at all points of the OGTT when compared with that of the nondiabetic control rats as represented in Table 1b and Figure 1b. To establish that intermittent fasting, starvation, and exercise enhanced glucose metabolism in diabetic rats, OGTTs were conducted in diabetic rats as represented in Table 1b and Figure 1b. At the beginning of intervention with intermittent fasting, starvation, and exercise, the level of blood glucose (indicated by the OGTT) was significantly lower than the diabetic group at 0, 30, 90, and 120 min after glucose loading. Although no significant changes were noticed in blood glucose level at all points of the OGTT when compared with that of the diabetic rats. This finding suggests glucotoxicity due to beta cell destruction, which was ameliorated by intermittent fasting, starvation, and exercise intervention.

### 4.2 Effect of intermittent fasting, starvation, exercise and honey on insulin tolerance test (ITT) in naïve, and streptozotocin‐induced diabetes in levels in male rats

**FIGURE 2:**
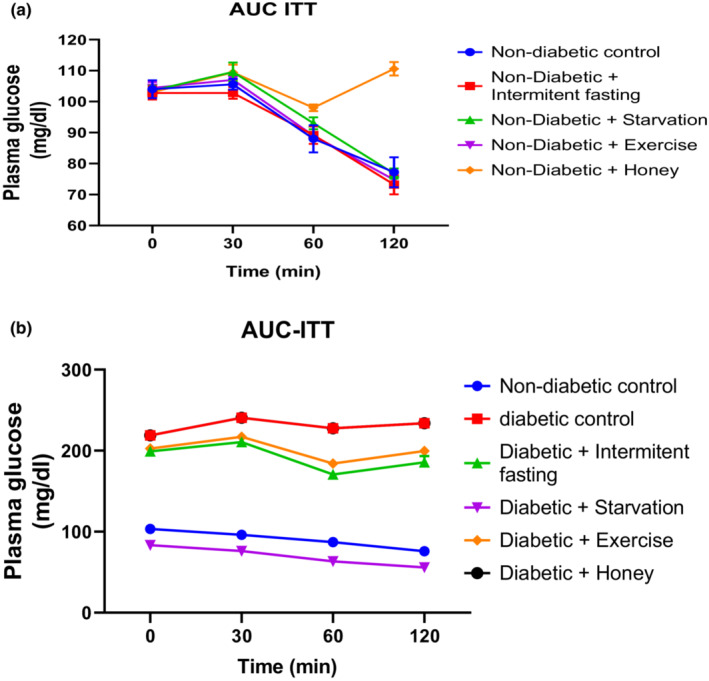
(a) Area under the curve (AUC) of insulin tolerance test on normal and different intervention protocols. (b) Insulin tolerance test on normal, diabetic rats and intervention protocol

The effect of intermittent fasting, starvation, exercise and honey on Insulin Tolerance Test (ITT) in naïve and streptozotocin‐induced Type 2 Diabetes Mellitus (T2DM) in male rats is shown in Table 2a and Figure 2a. This ITT was used to evaluate glucose metabolism in nondiabetic and diabetic rats. At the beginning of intervention with intermittent fasting, starvation, exercise, the level of glucose (indicated by the ITT) was not different across the groups at 0mins; however, the serum glucose level was significantly higher in the intermittent fasting, starvation, Exercise group than the control group at 30, 60, 90, and 120 min after glucose loading as represented in Table 2a and Figure 2a whereas, at 120 min, blood glucose levels were revealed to decreased in intermittent fasting and exercise group following an increased in rats treated with honey except in starvation were no changes was observed when compared to nondiabetic rats.

**TABLE 2:** (a) Insulin tolerance test on normal and intervention protocol. (b) Insulin tolerance test on normal, diabetic rats and intervention protocol

Groups	Plasma glucose (mg/dl)			
	0 min	30 min	60 min	120 min
**(a)**				
Normal diabetic control	104 ± 10.6	9.4 ± 8.91	88 ± 7.21	72.2 ± 6.32
ND + Intermittent fasting	102.8 ± 9.84	102.8 ± 11.2^*cd^	89.2 ± 7.43^*cd^	73.2 ± 4.78^*cd^
ND + Starvation	103.6 ± 9.88	109.6 ± 12.4^*bd^	93 ± 8.61*^bd^	76.8 ± 5.32^*bd^
ND + Exercise	104.4 ± 9.86	107.0 ± 12.6^*bcd^	89 ± 7.63*^bcd^	74.8 ± 5.71^*bcd^
ND + Honey	103.0 ± 9.86	109.4 ± 11.6^*^	98 ± 8.80*	110.6 ± 10.3^*^
**(b)**				
Normal diabetic control	103.3 ± 6.31	96.2 ± 3.64	87.3 ± 4.31	61.7 ± 3.30
Diabetic control (DC)	218.8 ± 13.63^*^	240.5 ± 10.81^*^	227.7 ± 12.13*	324.7 ± 3.69^*^
ND + Intermittent fasting	199 ± 5.36^ad^	210.5 ± 7.36^ad^	170.7 ± 9.31^ad^	274.8 ± 4.62^ad^
ND + Starvation	83.3 ± 4.73^abcd^	76.2 ± 5.10^abcd^	63.3 ± 3.20^abcd^	103.5 ± 4.14^abcd^
ND + Exercise	202.5 ± 7.83^ab^	217.2 ± 9.16^ab^	184 ± 6.34^ab^	264.8 ± 7.20^ab^
ND + Honey	216.9 ± 6.54	240.5 ± 11.41	226.4 ± 9.34	322.9 ± 8.52

Note

^a^ Showing glucose tolerance test on normal, diabetic rats and different intervention protocols Result represent Mean ± S.E.M. (*n* = 6) (One‐way ANOVA followed by Benferroni post hoc test). *P < 0.05, relative to controls. ^b^ *p* < 0.05 relative to intermittent fasting group; ^c^ *p* < 0.05 relative to Exercise group. ^d^ *p* < 0.05 relative to Honey group.

The results of the insulin tolerance test (ITT) of streptozotocin‐induced diabetic rats showed a significant increase in blood glucose levels at all points of the ITT when compared with that of the nondiabetic control rats as represented in Table 2b and Figure 2b. To confirm that intermittent fasting, starvation, and exercise enhanced glucose metabolism in diabetic rats, ITTs were conducted in diabetic rats as represented in Table 2b and Figure 2b. At the beginning of intervention with intermittent fasting, starvation, and exercise, the level of blood glucose (indicated by the ITT) was significantly lower than the diabetic group at 0, 30, 90, and 120 min after glucose loading. More specifically, starvation was revealed to exert more decrease in serum glucose level at all points of the ITT when compared to intermittent fasting and exercise.

### 4.3 Effect of intermittent fasting, starvation, exercise and honey on food intake and body weight in naïve, and streptozotocin‐induced diabetes in male rats

**FIGURE 3:**
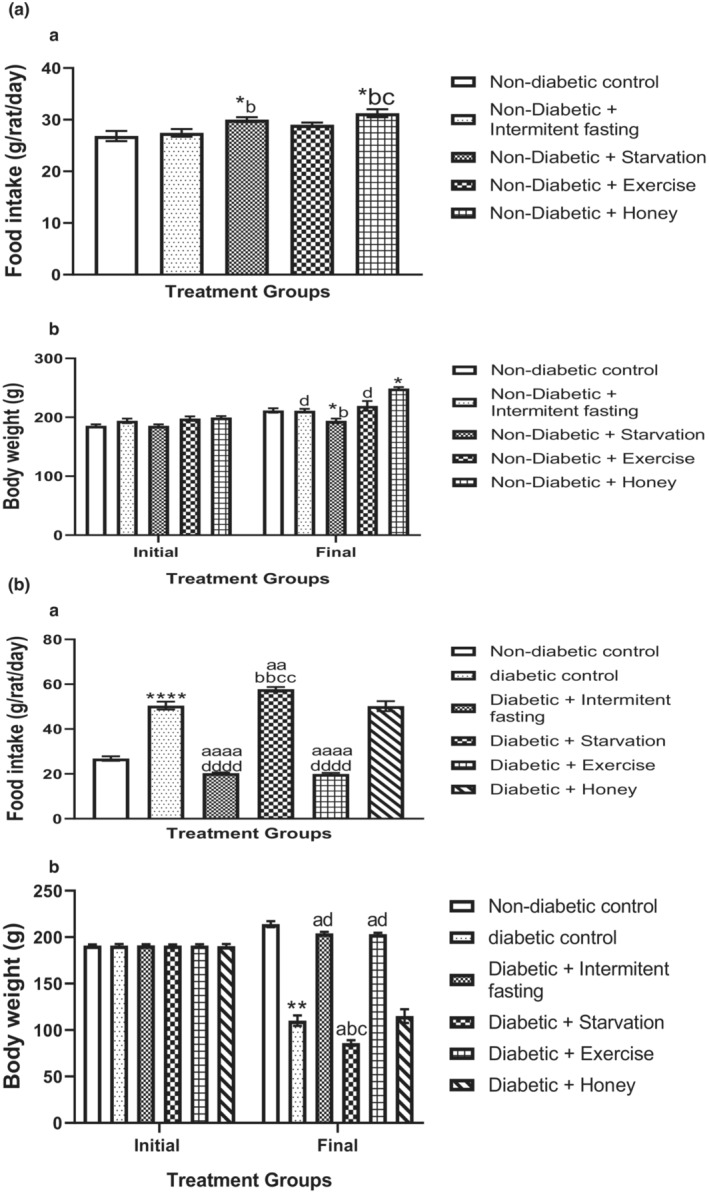
(A, B) Effect of intermittent fasting, starvation, exercise and honey on food intake (a) and body weight (b) in naïve male Wistar rats. Bars represent mean ± S.E.M. (*n* = 6) (one‐way ANOVA followed by Bonferroni post hoc test). (A) ^*^ *p* < 0.05 relative to controls. ^b^ *p* < 0.05 relative to intermittent fasting group; ^c^ *p* < 0.05 relative to exercise group. ^d^ *p* < 0.05, relative to honey group. (B) ^*^ *p* < 0.05, ^****^ *p* < 0.0001 relative to controls. ^a^ *p* < 0.05, ^aa^ *p* < 0.01, ^aaaa^ *p* < 0.001 relative to diabetic group. ^b^ *p* < 0.05, ^bb^ *p* < 0.01 relative to intermittent fasting group; ^c^ *p* < 0.05, ^cc^ *p* < 0.01 relative to Exercise group. ^d^ *p* < 0.001, ^dddd^ *p* < 0.0001 relative to honey group

Figure 3A,B show the effect of intermittent fasting, starvation, exercise and honey food intake and body weight in naïve and streptozotocin‐induced Type 2 diabetes Mellitus (T2DM) in male rats. As shown in Figure 3A, intervention with starvation and honey significantly (*p* > 0.001) increased food intake. Also, honey significantly increased body weight whereas starvation significantly decreased body weight relative to nondiabetic control, intermittent fasting, and exercise. Although no changes were observed in food intake and body weight in intermittent fasting and exercise when compared to nondiabetic control animals.

Changes in the amount of food intake as well as body weight were measured in the diabetic rats of experimental and nondiabetic control animals and expressed in Figure 3B. The amount of food intake was significantly (*p* < 0.001) increased whereas body weight was markedly (*p* < 0.001) decreased in the diabetic and starved rats relative to nondiabetic rats. Intermittent fasting and exercise intervention restored these changes to near‐normal. However, starvation and honey intervention with diabetic rats did not shows any significant changes in the amount of food intake.

### 4.4 Effect of intermittent fasting, starvation, exercise and honey on serum adipocytokines hormones (adiponectin, ghrelin, and irisin) in naïve, and streptozotocin‐induced diabetes in male rats

**FIGURE 4:**
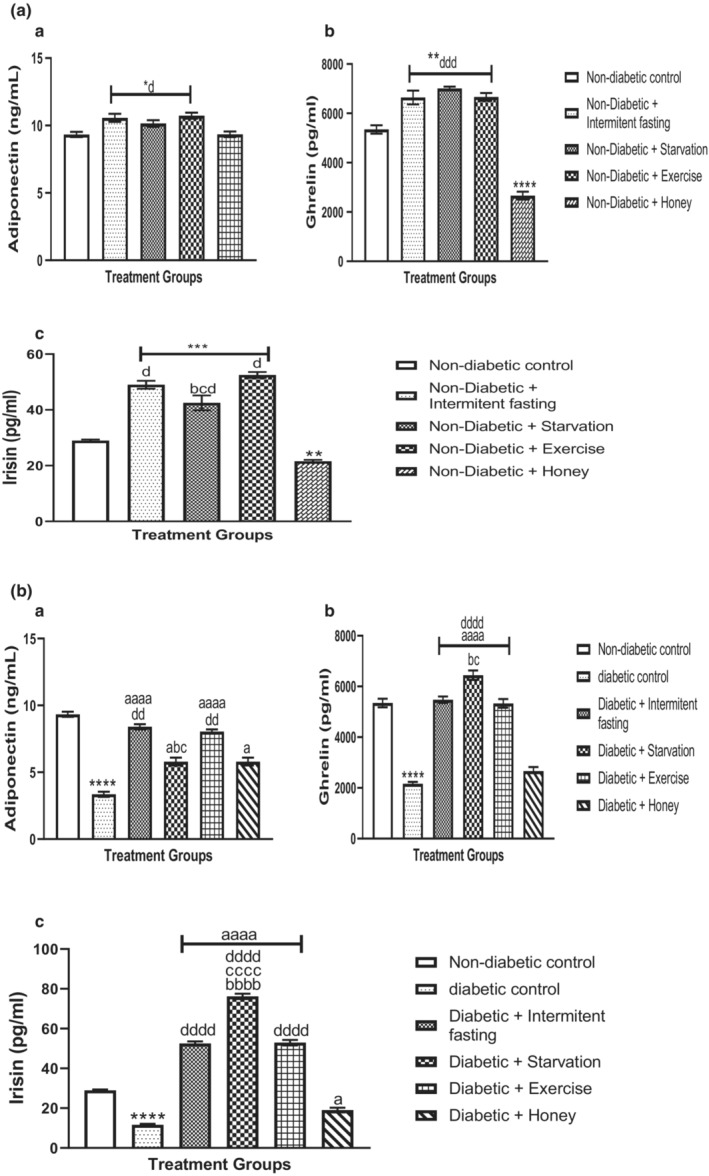
(A, B) Effect of intermittent fasting, starvation, exercise and honey on adiponectin (a), ghrelin (b), and irisin (c) activities in naïve male Wistar rats. Bars represent mean ± S.E.M. (*n* = 6) (one‐way ANOVA followed by Bonferroni post hoc test). (A) ^*^ *p* < 0.05, ^**^ *p* < 0.01, ^***^ *p* < 0.001, ^****^ *p* < 0.0001 relative to controls. ^b^ *p* < 0.05 relative to intermittent fasting group; ^c^ *p* < 0.05 relative to exercise group. ^d^ *p* < 0.05, ^dd^ *p* < 0.01, ^ddd^ *p* < 0.001 relative to honey group. (B) ^****^ *p* < 0.0001 relative to controls. ^a^ *p* < 0.05, ^aaaa^ *p* < 0.001 relative to diabetic group. ^bb^ *p* < 0.01, ^bbbb^ *p* < 0.0001 relative to intermittent fasting group; ^c^ *p* < 0.01, ^cccc^ *p* < 0.0001 relative to exercise group. ^dd^ *p* < 0.01, ^ddd^ *p* < 0.001, ^dddd^ *p* < 0.0001 relative to honey group

The effect of intermittent fasting, starvation, exercise and honey on serum adiponectin, ghrelin and irisin activities in naïve and streptozotocin‐induced Type 2 Diabetes Mellitus (T2DM) in male rats are shown in Figure 4A,B. As shown in Figure 4A, intervention with intermittent fasting and exercise significantly (*p* > 0.001) increased adiponectin (F [4, 25] = 7.689, *p* = 0.0003), ghrelin (F [4, 25] = 99.42, *p* < 0.0001), and irisin (F [4, 25] = 81.59, *p* < 0.0001). Starvation also significantly increased adiponectin, ghrelin, and irisin level but not to the extent of intermittent fasting and exercise when compared to nondiabetic control groups. No statistically significant changes were observed in serum adiponectin; although, a significant decrease was observed in irisin and ghrelin levels in rats treated with honey relative to nondiabetic controls. Furthermore, intermittent fasting and exercise intervention revealed marked significant changes in adiponectin, ghrelin, and irisin when compared with starvation and honey.

A statistically significant difference in the level of adiponectin, ghrelin, and irisin was evaluated in the diabetic rats’ serum of experimental and nondiabetic control animals and is expressed in Figure 4B. In the post hoc test, the concentration of serum adiponectin (F [5, 30] = 94.99, *p* < 0.0001), ghrelin (F [5, 30] = 127.6, *p* < 0.0001) (Figure 6B), irisin (F [5, 30] = 540.1, *p* < 0.0001) were markedly (*p* < 0.001) decreased in the serum of diabetic rats relative to nondiabetic rats. Intermittent fasting, starvation, exercise, and honey intervention restored these changes to near‐ normal. However, intermittent fasting and exercise intervention with diabetic rats shows more significant changes in the activities of adiponectin, ghrelin, and irisin when compared to starvation and honey.

### 4.5 Effect of intermittent fasting, starvation, exercise and honey on serum oxidative glucose metabolic enzymes/mitochondria dehydrogenase (ICDH, SDH, G6PDH, and LDH) in naïve and streptozotocin‐induced diabetes in male rats

**FIGURE 5:**
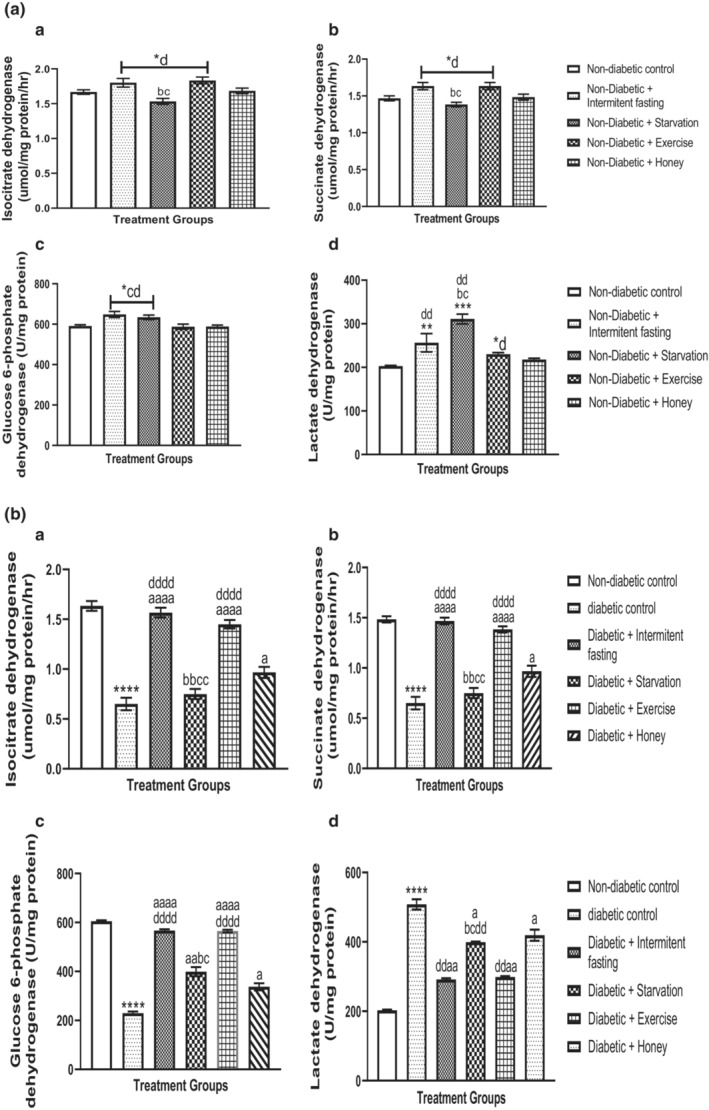
(A, B) Effect of intermittent fasting, starvation, exercise and honey on serum isocitrate dehydrogenase (ICDH) (a), succinate dehydrogenase (SDH), glucose‐6‐phosphate dehydrogenase (G6PDH) and lactate dehydrogenase (LDH) (b) activities in naïve male Wistar rats. Bars represent mean ± S.E.M. (*n* = 6) (one‐way ANOVA followed by Bonferroni post hoc test). (A) ^*^ *p* < 0.05, ^**^ *p* < 0.01, ^***^ *p* < 0.001 relative to controls. ^b^ *p* < 0.05 relative to intermittent fasting group; ^c^ *p* < 0.05 relative to exercise group. ^d^ *p* < 0.05, ^dd^ *p* < 0.01, relative to honey group. (B) ^****^ *p* < 0.0001 relative to controls. ^a^ *p* < 0.05, ^aa^ *p* < 0.01, ^aaaa^ *p* < 0.001 relative to diabetic group. ^b^ *p* < 0.05, ^bb^ *p* < 0.01 relative to intermittent fasting group; ^c^ *p* < 0.05, ^cc^ *p* < 0.01 relative to exercise group. ^d^ *p* < 0.05, ^dd^ *p* < 0.01, ^dddd^ *p* < 0.0001 relative to honey group

The effect of intermittent fasting, starvation, exercise and honey on serum oxidative glucose metabolic enzymes/mitochondria dehydrogenase (ICDH, SDH, G6PDH, and LDH) in naïve and streptozotocin‐induced Type 2 Diabetes Mellitus (T2DM) in male rats are shown in Figure 5A,B. As shown in Figure 5A, intervention with intermittent fasting and exercise significantly (*p* > 0.001) increased ICDH (F [4, 25] = 6.497, *p* = 0.0010) (Figure 5a), SDH (F [4, 25] = 7.094, *p* = 0.0006), G6PDH (F [4, 25] = 6.966, *p* = 0.0007), and LDH (F [4, 25] = 15.19, *p* < 0.0001). Starvation decreased ICDH, SDH, and LDH levels and increased G6PDH when compared to intermittent fasting and exercise; although no significant changes were observed in G6PDH when compared to honey and nondiabetic control groups. No statistically significant changes were observed in ICDH, SDH, LDH, and G6PDH levels in rats treated with honey relative to nondiabetic controls. More so, intermittent fasting and exercise intervention revealed marked significant Changes in ICDH, SDH, LDH, and G6PDH levels when compared with starvation and honey.

Changes in the level of ICDH, SDH, LDH, and G6PDH were evaluated in the diabetic rats’ liver of experimental and nondiabetic control animals as expressed in Figure 5B. The diabetic rat serum was revealed to depicted a marked (*p* < 0.001) reduction in ICDH (F [5, 30] = 69.67, *p* < 0.0001), SDH (F [5, 30] = 67.77, *p* < 0.0001), and G6PDH (F [5, 30] = 203.1, *p* < 0.0001) activities and a marked (*p* < 0.001) increased in LDH (F [5, 30] = 144.2, *p* < 0.0001) activity. The changes of ICDH, SDH, G6PDH, and LDH activities were reverted to the normal range in the serum of intermittent fasting and exercise intervention with diabetic rats. Starvation intervention compared with diabetic control rats did not illustrate any marked differences in ICDH and SDH activities but was observed to increased G6PDH and LDH, although this elevation and that of the honey intervention were not to the level of intermittent fasting and exercise intervention.

### 4.6 Effect of intermittent fasting, starvation, exercise and honey on glycolytic enzymes/nonmitochondrial enzymes (G6Pase, F1,6BPase, HKase, and PKase) in naïve and streptozotocin‐induced diabetes in male rats

**FIGURE 6:**
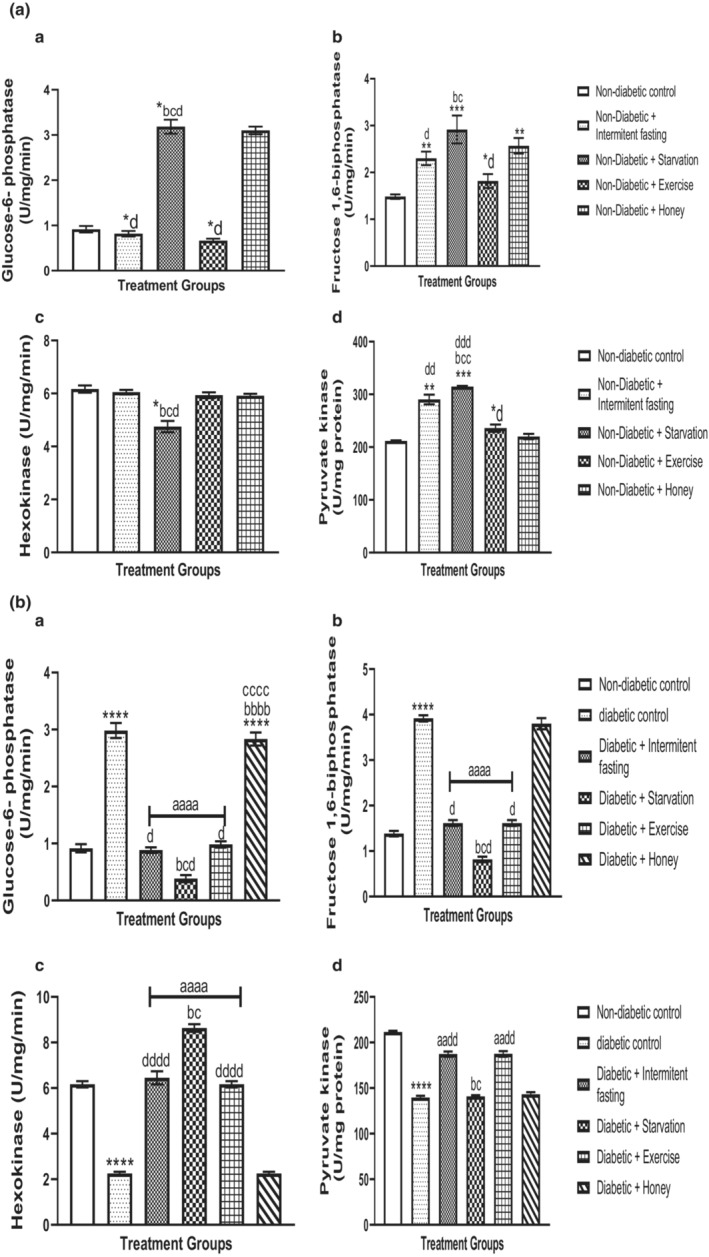
(A, B) Effect of intermittent fasting, starvation, exercise and honey on glucose‐6‐phosphatase (G6Pase) (a), fructose‐1,6 biphosphatase (F1,6BPase) (b), hexokinase (HKase) (c) and pyruvate kinase (PKase) (d) activities in naïve male Wistar rats. Bars represent mean ± S.E.M. (*n* = 6) (one‐way ANOVA followed by Bonferroni post hoc test). (A) ^*^ *p* < 0.05, ^**^ *p* < 0.01, ^***^ *p* < 0.001 relative to controls. ^b^ *p* < 0.05 relative to intermittent fasting group; ^c^ *p* < 0.05 relative to exercise group. ^d^ *p* < 0.05, ^dd^ *p* < 0.01 relative to honey group. (B) ^****^ *p* < 0.0001 relative to controls. ^aa^ *p* < 0.01, ^aaaa^ *p* < 0.001 relative to diabetic group. ^b^ *p* < 0.05 relative to intermittent fasting group; ^c^ *p* < 0.05 relative to exercise group. ^d^ *p* < 0.05, ^dd^ *p* < 0.01, ^dddd^ *p* < 0.0001 relative to honey group

The effect of intermittent fasting, starvation, exercise and honey on liver glycolytic enzymes/non mitochondrial enzymes (G6Pase, F1,6BPase, HKase, and PKase) in naïve and streptozotocin‐induced Type 2 Diabetes Mellitus (T2DM) in male rats are shown in Figure 6A,B. As shown in Figure 6A, intervention with intermittent fasting and exercise significantly (*p* > 0.001) decreased G6Pase (F [4, 25] = 6.497, *p* = 0.0010) and increased F1,6B Pase (F [4, 25] = 7.094, *p* < 0.0001) and PKase (F [4, 25] = 6.966, *p* < 0.0001); whereas no significant changes were observed in HKase (F [4, 25] = 15.19, *p* < 0.0001). Starvation markedly decreased G6Pase, F1,6BPase, Pkase level, and decreased HKase when compared to intermittent fasting and exercise. No statistically significant changes were observed in HKase and PKase level in rats treated with honey relative to nondiabetic controls. Furtherly, intermittent fasting and exercise intervention revealed marked significant Changes in G6Pase, F1,6BPase, Pkase level, and decreased HKase level when compared with starvation and honey.

Changes in the level of G6Pase, F1,6BPase, HKase, and Pkase were evaluated in the diabetic rats’ liver of experimental and nondiabetic control animals as expressed in Figure 6B. The diabetic rat liver were shown to depicted a marked (*p* < 0.001) reduction in G6Pase (F [5, 30] = 69.67, *p* < 0.0001), F1,6BPase (F [5, 30] = 67.77, *p* < 0.0001) and HKase and PKase (F [5, 30] = 203.1, *p* < 0.0001) activities shows marked (*p* < 0.001) increased (F [5, 30] = 144.2, *p* < 0.0001) activity. The changes of G6Pase, F1,6BPase, HKase, and Pkase activities were reverted more to the normal range in the liver of starvation as well as in the intermittent fasting and exercise intervention with diabetic rats. Honey intervention compared with diabetic control rats did not illustrate any marked differences in G6Pase, F1,6BPase, HKase, and Pkase activities but were observed to increase G6Pase, F1,6BPase; whereas no changes were observed in the HKase and Pkase.

## 5 DISCUSSION

In the current investigation, STZ‐induced diabetic rats had reduced glucose tolerance and insulin sensitivity, as demonstrated by higher blood glucose and insulin levels at all points of OGTT and ITT (Abdulwahab et al., 2021; Oza & Kulkarni, 2018). The STZ induces type 2 diabetes, as well as reduced glucose tolerance and insulin resistance, according to the Mahmoud et al. (2017).

Although insulin levels may be normal or even elevated in some diabetic patients, most tissues are unable to use glucose, resulting in hyperglycemia. Glucose intolerance is the medical term for this. One of the most prominent procedures for evaluating glucose intolerance is the oral glucose tolerance test (OGTT). It looks into problems with blood glucose regulation or glucose homeostasis. The blood glucose levels of diabetic rats were considerably raised after consumption of glucose during an OGTT in this investigation (Lodhi and Kori, 2021; Germoush et al., 2019). With intermittent fasting, starvation, and exercise intervention, the concentration of blood glucose in diabetic rats was elevated to a peak after 30 min, and then restored to fasting blood glucose ranges after 60 and 120 min. Untreated diabetic rats, on the other hand, exhibited greater blood glucose levels at 30 and even 120 min. When honey‐treated diabetic rats were compared to untreated diabetic rats, it was shown that glucose levels remained higher. Intermittent fasting, starving, and exercise were found to assist increase glucose tolerance by lowering glucose absorption from the intestine, enhancing insulin sensitivity, and boosting insulin action on diverse tissues for glucose uptake, according to the findings of Albosta and Bakke (2021) and Dwaib et al. (2021).

Weight loss, muscle wasting, excessive hair loss, scaling, cataract, increased food and water consumption, polyuria, dehydration, and other symptoms are all observed in diabetic rats. In this work, the bodyweight of STZ‐induced diabetic rats was dramatically lowered. Because diabetic rat cells may be unable to use glucose for energy production due to decreased insulin action or secretion, this could be explained by higher consumption of fat and protein. Increased protein catabolism to generate amino acids for gluconeogenesis also leads to muscle waste and weight loss (Srinivasan et al., 2014). In the current study, the body weight of diabetic rats was dramatically lowered. This drop could be due to structural protein breakdown, which contributes to weight gain (Mahajan et al., 2020). In STZ‐induced experimental DM, weight loss is associated to increased tissue protein breakdown and muscle degeneration (Mahajan et al., 2020).

The amount of food ingested by control and experimental rats was also recorded or quantified on a daily basis in this study. Food consumption rose dramatically in diabetic rats, which could be due to impaired glucose utilization by tissues, resulting in a high amount of glucose excretion through urine, which produces a persistent stimulus to eat more food. In the intermittent and activity groups, diabetic rats were less likely to lose weight and eat more food. This could be due to the fact that intermittent fasting and exercise help to manage blood sugar levels (Albosta & Bakke, 2021; Spezani et al., 2020). By decreasing calorie intake and resetting the metabolism, intermittent fasting can assist to reduce obesity and, as a result, insulin resistance. Furthermore, greater AMP‐activated protein kinase (AMPK) activation has been demonstrated to promote healthy aging and a reduction in chronic disease through energy/nutrient depletion (such as caloric restriction) (Burkewitz et al., 2016). Reduced energy intake, such as that obtained through intermittent fasting, should result in long‐term reductions in insulin production, as seen in this study, as well as increased levels of AMPK, which is thought to play a role in improved insulin sensitivity and glucose homeostasis, as seen in this study. (Larson‐Meyer et al., 2006) discovered that in overweight, glucose‐tolerant persons, a 25% reduction in calories, either by diet alone or diet combined with exercise, enhanced insulin sensitivity and reduced cell sensitivity.

Several obesity studies, on the other hand, have found that humans have a hard time sticking to a daily calorie restriction for long periods of time (Anton et al., 2017). Intermittent fasting, on the other hand, has a higher compliance rate and has been shown to help obese people improve metabolic risk factors, body composition, and weight loss (Albosta & Bakke, 2021; Anton et al., 2017; Spezani et al., 2020). The shift in the body's main fuel source during fasting from glucose to fatty acids and ketones has been related to these favorable outcomes. (Anton et al., 2017).

We measured serum adiponectin and ghrelin levels in diabetic rats to better understand the physiological mechanisms by which intermittent fasting and exercise exert their therapeutic intervention on serum glucose and insulin levels. Adipokines are involved in energy homeostasis and the regulation of glucose and lipid metabolism, immunity, neuroendocrine function, insulin‐sensitization, anti‐inflammatory, and antiatherogenic function, and cardiovascular function (Duszka et al., 2021; Dwaib et al., 2021; Liang et al., 2021; Di Sessa et al., 2019; Spezani et al., 2020;). In research, adiponectin was found to affect insulin sensitivity in diabetic mice (Saad et al., 2015). Adiponectin levels are low in persons with obesity, type 2 diabetes, and coronary artery disease (Looker et al., 2004; Raji et al., 2004). In this investigation, diabetic rats experienced a considerable drop in serum adiponectin, as previously reported (Ahmed et al., 2012; Mahmoud et al., 2013). Lower serum levels of adiponectin and ghrelin have been associated with insulin resistance, poor insulin sensitivity, and the genesis of obesity and type 2 diabetes (Li et al., 2020; Statnick et al., 2000). For 4 weeks, diabetic rats who fasted intermittently and exercised had greater blood levels of adiponectin and ghrelin (Ouerghi et al., 2021; Stensel, 2010). Improvements in glucose tolerance, insulin sensitivity, hepatic glucose production, and peripheral glucose uptake were associated to this (Polito et al., 2020; Stensel, 2010). According to current data, ghrelin may play a function in metabolic syndrome (Ukkola, 2009). In a range of pathophysiological situations, such as obesity, type 2 diabetes, and other metabolic abnormalities, ghrelin concentrations have been demonstrated to be lowered (Barazzoni et al., 2007; Poykko et al., 2003). Insulin has been proven to decrease ghrelin release in healthy normal‐weight and overweight adults (St‐Pierre et al., 2007; Weickert et al., 2008). Hyperinsulinemia with simultaneous hyperglycemia has no influence on plasma ghrelin at concentrations seen in insulin‐resistant patients, according to a prior study, but only at pharmacological insulin doses. Because ghrelin has been found to drive adipogenesis in vitro, the decline in adiponectin could be attributable to a drop in ghrelin levels (Mano‐Otagiri et al., 2009). By suppressing gluconeogenesis and boosting lipid oxidation, adiponectin has been demonstrated to increase AMP‐activated protein kinase (AMPK), resulting in better insulin sensitivity and glucose metabolism regulation (Yamauchi et al., 2002). Adiponectin also suppresses hepatic gluconeogenesis by lowering the expression of glucose‐6‐phosphatase and phosphoenolpyruvate carboxylase, lowering hepatic glucose production (Yamauchi et al., 2002). Through these processes, adiponectin and ghrelin contribute to enhanced insulin‐induced signal transduction and hence improved insulin sensitivity (Ouerghi et al., 2021; Yamauchi et al., 2002).

Irisin, a new adipocytokine, is released, activated, and transported to a variety of tissues and organs to carry out its physiological tasks. It can, for example, improve insulin resistance, boost uncoupling protein‐1 expression, convert white fat into brown fat with catabolic properties, increase energy consumption and glucose utilization, and coordinate the treatment of metabolic illnesses like obesity and type 2 diabetes (Jung et al., 2017; Rizk et al., 2016; Xuan et al., 2020).

As a result, unlike starvation, exercise and intermittent fasting can improve insulin resistance and have a modest hypoglycemic impact, as revealed in our work. This could be due to exercise increasing irisin secretion in skeletal muscle (Liu et al., 2021; Sousa et al., 2021; Xuan et al., 2020). Our findings revealed that STZ‐induced diabetic rats had lower irisin levels than non‐diabetic controls, which was consistent with and similar to the findings of most previous studies in animals and humans when compared to nondiabetic controls (Choi et al., 2013; Elizondo‐Montemayor et al., 2019; Liu et al., 2013; Moreno‐Navarrete et al., 2013; Yan et al., 2014; Zhang et al., 2016; Xuan et al., 2020). When diabetic rats were compared to nondiabetic control rats, non‐pharmacological therapies such as intermittent fasting and exercise were observed to generate an increase in serum irisin. Intermittent fasting/exercise‐related elevated serum irisin has been connected to improved metabolic health, insulin signaling, glucose homeostasis, and other glycemic profile in animal STZ‐models of diabetes, making it a prospective target in the management of metabolic diseases.

The glycolysis pathway, which starts with hexokinase phosphorylating glucose to glucose 6‐phosphate, is the core of cellular metabolism (HK). In energy metabolism, the isoenzyme HK plays a crucial function. In mammalian cells, hexokinases (HKs) are four isoforms of hexokinases that are involved in glucose oxidation (Wilson, 1995). The activity of HK I‐III is regulated by the cell's glucose 6‐phosphate concentration, which acts as a feedback inhibitor. Insulin, glucagon, and glucokinase regulatory protein regulate the activity of HK‐IV, commonly known as glucokinase, which has a low affinity for glucose yet phosphorylates it predominantly (Collier & Scott, 2004). HK‐I and HK‐IV are expressed more in the liver than the other HKs. Previous research has shown that liver HK is important for glucose consumption and glycogen production (Postic, 2001), and that its activity is decreased in diabetes. The liver activity of diabetic rats was shown to be significantly lower in this investigation. A decrease in insulin sensitivity and an increase in insulin resistance could be to blame. After intervention with intermittent fasting and exercise, the HK activity in the liver of diabetic rats was dramatically increased. Intermittent fasting and exercise may have activated insulin sensitivity for glucose reuptake by the cells, resulting in this rise. Intermittent fasting and exercise enhanced glucose metabolism and glucose homeostasis by boosting HK activity in the liver.

Pyruvate kinase (PK) transforms phosphoenolpyruvate to pyruvate and generates ATP. L (liver‐type), R (red blood cell‐type), M1 (muscle‐type), and M2 (muscle‐type) are the four isoforms of PK (muscle‐type). Yamada and Noguchi (1998) showed that PK‐L is expressed greatest in the liver and lowest in the kidneys, pancreatic b‐cells, and small intestine, whereas PK‐R is exclusively present in red blood cells. PK‐M1 is present in the brain, heart, and skeletal muscle, while PK‐M2 is found in other tissues (Noguchi et al., 1991). In persons with diabetes, reduced PK activity may be the reason for impaired glucose metabolism and ATP generation. The current study found a considerable reduction in PK activity in the livers of STZ‐induced diabetic rats, resulting in decreased glycolysis and enhanced gluconeogenesis. Earlier research had yielded similar findings (Palsamy & Subramanian, 2009; Prasath & Subramanian, 2011; Srinivasan et al., 2014). The PK activity in the livers of diabetic rats was recovered to near‐normal levels with intermittent fasting and exercise.

The enzyme G6Pase (glucose‐6‐phosphatase) is essential for glucose homeostasis. Bouché et al. (2004) identified it largely in the liver and kidney, where it aids in glucose production during famine or prolonged fasting, as well as diabetes mellitus. G6Pase is engaged in the glycogenolysis and gluconeogenesis pathways’ dephosphorylation step, where glucose‐6‐phosphate is transformed to free glucose. This enzyme, which is connected to the glucose‐6‐phosphate transporter, hydrolyzes glucose‐6‐phosphate into glucose and phosphate in the endoplasmic reticulum (Chou et al., 2002). G6Pase is activated by cAMP, whereas insulin inhibits it. Similar to prior investigations, the current study discovered a considerable increase in G6Pase activity in the liver of STZ‐induced diabetic rats (Palsamy & Subramanian, 2009; Prasath & Subramanian, 2011; Srinivasan et al., 2014). Intermittent fasting and exercise brought G6Pase activity back to near‐normal levels in diabetic mice. Fructose‐1,6‐bisphosphatase (F1,6BP) is a rate‐limiting enzyme in the gluconeogenic pathway that dephosphorylates fructose‐1,6‐bisphosphate to fructose‐6‐phosphate. It is usually present in the liver and kidney, but it can also be found in the b‐cells of the pancreas. In this investigation, the activity of F1,6BP in the liver of STZ‐induced diabetic rats was found to be considerably higher.

This result is in line with prior findings (Palsamy & Subramanian, 2009; Prasath & Subramanian, 2011; Srinivasan et al., 2014). Increased F1,6BP activity may be a mechanism to initiate endogenous glucose production from glycerol via gluconeogenesis during diabetes (Nurjhan et al., 1992). Intermittent fasting and exercise drastically lowered F1,6BP activity in diabetic rats’ livers, restoring glucose homeostasis by limiting gluconeogenesis via gluconeogenic substrates while blocking direct impacts on glycolysis, glycogenolysis, and the citric acid cycle.

During anaerobic glycolysis, which occurs both in the cytosol and in the mitochondria, LDH converts pyruvate to lactate to provide energy (Bouché et al., 2004; Kavanagh et al., 2004; Kavanagh et al., 2004). H (heart‐type) and M (muscle‐type) are the two subunits of LDH, and their synthesis is controlled by two distinct genes. Glucose, insulin, and NADH limit LDH activity, whereas cytosolic ATP, Ca2+, and mitochondrial membrane potential boost it (Ainscow et al., 1999). Reduced LDH activity in tissues may be needed to confirm that glycolysis produces a high ratio of NADH and pyruvate, which is oxidized by mitochondria. In this work, the activity of LDH was observed to be considerably higher in the livers of STZ‐induced diabetic rats. Similar findings have been seen in other studies (Palsamy & Subramanian, 2009; Prasath & Subramanian, 2011). Diabetes‐related increases in LDH activity may disrupt glucose metabolism and reduce insulin sensitivity. The activity of LDH in the liver of diabetic rats was returned to near‐normal by manipulating the ratio of NADH and pyruvate with intermittent fasting and exercise. As a result, the process of glucose (pyruvate) oxidation in the mitochondria is improved.

G6PDH is a pentose phosphate pathway regulator that creates NADPH, which is needed to restore reduced glutathione from oxidized glutathione.

According to an earlier study, NADPH produced by G6PDH is essential for the generation of reactive oxygen species (ROS) such as superoxide and nitric oxide radicals in hepatic and extrahepatic tissues, as well as their eradication by catalase and glutathione peroxidase (GPx) (Park et al.,^2006^). Glutathione levels have been associated to reduce oxidative stress and G6PDH activity (Dora et al., 2021; Nóbrega‐Pereira et al., 2016). In this work, the activity of G6PDH in the liver of diabetic rats was found to be considerably lower. This finding is in line with earlier research (Palsamy & Subramanian, 2009; Prasath & Subramanian, 2011; Srinivasan et al., 2014). The reduced activity of G6PDH could possibly contribute to the advancement of diabetes complications. With intermittent physical exercise, G6PDH activity in diabetic rats was considerably increased to near‐normal levels. Furthermore, both intermittent fasting and exercise treatments boosted hexokinase and pyruvate kinase activity in the diabetic rats’ livers while lowering glucose‐6‐phosphatase and fructose‐1,6‐biphosphatase. Diabetes increases the rate of glycogenolysis and gluconeogenesis, resulting in higher hepatic glucose production (Raju et al., 2001). Hexokinase activity was found to be lower and glucose‐6‐phosphatase activity was shown to be higher in previous studies, resulting in lower liver glycogen and hyperglycemia (Ahmed et al., 2012; Grover et al., 2000). Increased insulin production with a matching rise in insulin resistance, which activates the glycogenolytic and gluconeogenic pathways, is another mechanism contributing to a decrease in liver glycogen (Mahmoud et al., 2015; Pari & Murugan, 2005).

The drop in SDH activity generated by STZ‐induced oxidative stress suggests a decrease in succinate to fumarate conversion, reflecting a decrease in oxidative metabolism. The synthesis of fumarate is increased when phosphoenolpyruvate is diverted during a stressful scenario, resulting in SDH product inhibition (Rajeswarareddy et al., 2012). SDH activity may be reduced in diabetic rats’ tissues due to enzymatic failure caused by lipid peroxidation activation. This could be owing to an excess of free radicals created in response to the harmful effects. Diabetic rats on a non‐pharmacological intermittent fasting and exercise program had higher SDH activity than diabetic rats on a pharmacological intermittent fasting and exercise regimen. The antioxidant‐boosting benefits of intermittent fasting and exercise could be to blame for this increase (Allen et al., 2020; Nurmasitoh et al., 2018; Shahandeh et al., 2013). Furthermore, higher SDH activity in diabetic rats during intermittent fasting and exercise suggests that the TCA cycle is more efficient at using energy‐producing intermediates. Isocitrate dehydrogenase (ICDH) catalyzes the oxidative decarboxylation of isocitrate to ‐ketoglutarate, which requires either NAD+or NADP+to create NADH and NADPH, respectively (Rajeswarareddy et al., 2012). NADPH is necessary for the operation of the NADPH‐dependent thioredoxin system and the regeneration of reduced glutathione (GSH) by glutathione reductase, both of which are vital in the protection of cells against oxidative damage (Rajeswarareddy et al., 2012). As a result, during oxido‐nitrergic stress, ICDH could operate as an antioxidant. By providing NADPH for GSH synthesis, ICDH protects mitochondrial, and cytosolic oxidative damage (Rajeswarareddy et al., 2012). As a result of ICDH damage, the equilibrium between oxidants and antioxidants may be disrupted, resulting in a prooxidant state. In STZ‐induced diabetes rats, the activity of isocitrate dehydrogenase (ICDH) was assessed, and it was found to be considerably lower in the diabetic group than in the nondiabetic control group. Rajeswarareddy et al. (2012) published similar findings, indicating that diabetic group mitochondrial ICDH activity was lower than nondiabetic control group. The glycation of ICDH can prevent it from performing its function. Glycation aids the inactivation of ICDH by reactive oxygen species. After non‐pharmacological treatments with intermittent fasting and exercise, the activity of ICDH was normalized when compared to the diabetes control group. This could be attributed to intermittent fasting and exercise's antioxidant‐enhancing or mediating activity in reducing diabetes complications.

## 6 CONCLUSION

In conclusion, our findings show for the first time that nonpharmacological therapeutic regimens such as intermittent fasting and exercise improve insulin sensitivity and glucose tolerance in STZ‐induced type 2 diabetic rats by maintaining insulin signaling and glucose homeostasis, whereas starvation had more hypoglycemic effects, resulting in increased weight loss. The honey‐treated rats show higher diabetes‐related symptoms. Intermittent fasting and exercise boosted peripheral glucose absorption, decreased hepatic glucose production, regulated glucose metabolic enzymes, and raised the activity of liver glycolytic enzymes in diabetic rats. In diabetic rats, intermittent fasting, and exercise enhanced serum adipocytokines levels. These findings imply that adipokines modulate glycolytic/nonmitochondrial enzymes and glucose metabolic/mitochondrial dehydrogenase to mediate the antidiabetic effects of intermittent fasting and exercise.

## CONFLICT OF INTEREST

There were no conflict of interest revealed by the authors.

## AUTHOR CONTRIBUTIONS

Conceptualization E.A.C., N.E.K; data curation, writing original draft preparation. E.A.E., O.M.O. and B.O.O.; review and editing O.M.O., N.E.K., and E.V.; supervision N.E.K.; validation N.E.K.; funding acquisition E.A.C., B.O.O., and O.M.O. All authors have read and agreed to the publishing of the manuscript.

## ETHICAL APPROVAL

The Ethical Review Committee of Delta State University gave their approval to perform this study on 09/11/2021, with the reference number REC/FBMS/DELSU/21/121. The Delta State University Ethical Review Committee (DSUERC) guarantees that all institutional guidelines and regulations are followed, as well as that all adverse events are reported to the DSUERC as soon as possible. The research could not be changed without DSUERC's prior consent.

## CONSENT TO PARTICIPATE

Not applicable.

## CONSENT FOR PUBLICATION

All authors gave their consent for the article to be published.

## CODE AVAILABILITY

Not applicable.
